# Immune-Checkpoint Inhibition in the Treatment of Gastro-Esophageal Cancer: A Closer Look at the Emerging Evidence

**DOI:** 10.3390/cancers13235929

**Published:** 2021-11-25

**Authors:** Koosha Paydary, Natalie Reizine, Daniel V. T. Catenacci

**Affiliations:** Section of Hematology/Oncology, Department of Medicine, University of Chicago Medical Center and Biological Sciences, Chicago, IL 60637, USA; koosha.paydary@uchospitals.edu (K.P.); nreizi2@uic.edu (N.R.)

**Keywords:** immune-checkpoint inhibitor, gastroesophageal cancer, programmed death ligand-1

## Abstract

**Simple Summary:**

Gastroesophageal cancers (GEC) constitute a common group of tumors that vary highly in incidence and treatment response. Although chemotherapy is the mainstay of treatment, immunotherapy with immune-checkpoint inhibitors (ICI) is a novel treatment modality for this type of cancer. To date, several studies have evaluated the safety and efficacy of ICIs for the treatment of GEC. The role of ICIs in the treatment of GEC is rapidly evolving. In the US, ICIs have established indications for second-line treatment of microsatellite unstable tumors, while their use in third-line settings was recently withdrawn. Notably, the use of ICIs for first-line therapy of GEC includes high PD-L1 expressing tumors, irrespective of HER2 status, and in the adjuvant setting after neoadjuvant chemoradiotherapy in select patients. In this review, we outline the results of these studies for the third-line, second-line, first-line, and peri-operative treatment of GEC.

**Abstract:**

To date, several trials have evaluated the safety and efficacy of immune-checkpoint inhibitors (ICI) for the treatment of gastroesophageal cancers (GEC). In the US, ICIs have established indications for second-line treatment of microsatellite unstable tumors, while their use in third-line settings was recently withdrawn. Notably, the use of ICIs for first-line therapy of GEC is rapidly evolving, which currently includes high PD-L1 expressing tumors, irrespective of HER2 status, and in the adjuvant setting after neoadjuvant chemoradiotherapy in select patients. In this article, we review the results of studies that have evaluated the utility of ICI in the third-line, second-line, first-line, and peri-operative treatment settings of GECs. Considerations should be made before making any cross-trial comparisons since these trials vary in chemotherapy backbone, anatomical and histological eligibility, biomarker assessment, PD-L1 diagnostic antibodies, and definition of PD-L1 positivity. Regardless, the totality of the data suggest that first-line ICI use may most benefit GEC patients with high PD-L1 combined positivity score (CPS) ≥5 or ≥10, irrespective of histology or anatomy. Moreover, although PD-L1 by CPS has a good negative predictive value for significant benefit from ICIs, it has a low positive predictive value. Therefore, there is a pressing need to identify better biomarkers to predict benefit from ICIs among these patients.

## 1. Introduction

Gastro-esophageal cancers (GEC) constitute a heterogeneous group of tumors that vary highly in incidence, histology, molecular biology, and response to treatments [1,2]. There are many GEC subtypes, including esophageal squamous cell carcinoma (esoSCC), gastro-esophageal junction (GEJ) adenocarcinoma (AC), and distal gastric AC. With respect to treatment, either a combined modality chemoradiation therapy (CRT) or perioperative therapy are the standard neoadjuvant approaches in treating locally advanced GEC [3,4,5,6]. In the metastatic setting, a combination regimen with a backbone of a platinum plus a fluoropyrimidine is the preferred first-line palliative treatment; however, prognosis remains poor, with a median overall survival (mOS) of ~12 months with this approach [7,8,9,10].

Recently, molecular profiling, and in particular the use of next-generation sequencing (NGS), has led to the identification of a number of potential therapeutic targets in GEC [11,12,13,14]. In 2010, HER2-directed therapy combined with chemotherapy backbone was approved as the standard of care for HER2-positive metastatic GEC [15]. In the ToGA study (Trastuzumab for gastric cancer), median OS was 13.8 months in those assigned to trastuzumab plus chemotherapy compared with 11.1 months in those assigned to chemotherapy alone, and in patients with a higher threshold of HER2 positivity and now the clinical standard (IHC2+/FISH+ or IHC3+), the survival benefits were more pronounced [15]. Since then, the landscape of HER2-directed therapy, as well as role of other actionable molecular subgroups, has been evolving [16,17,18,19,20,21,22]. Furthermore, this growing inter-individual molecular heterogeneity of patients is further complicated by the appreciation of intra-individual molecular heterogeneity through space and over time, offering further improvements for personalized treatments [23,24,25].

Immunotherapy with immune-checkpoint inhibitors (ICIs) offers an exciting avenue for the treatment of GEC. To date, several trials have evaluated the efficacy and safety of ICI for the treatment of GEC [26,27]. In these trials, PD-L1 status has been reported using combined positive score (CPS) or tumor positive score (TPS): while CPS is defined as the number of PD-L1 staining cells (tumor cells, lymphocytes, macrophages, etc.,) divided by the total number of viable tumor cells, TPS only considers PD-L1 staining tumor cells in the nominator. For adenocarcinomas, the incidence of TPS ≥ 1 ranges from ~10–25%, and CPS ≥ 1 ranges from ~55–85%, while the incidence of CPS ≥ 5 ranges from 35–60% and CPS ≥ 10 ranges from 15–45% depending on the assays used and the cohorts studied. According to current NCCN guidelines, ICIs are recommended in the metastatic setting for first-line use in high PD-L1 expressing tumors and second-line use in MSI-high tumors. Although pembrolizumab was previously indicated for third-line or higher setting of PD-L1 positive (CPS ≥ 1) tumors, FDA’s Oncologic Drugs Advisory Committee (ODAC) recently voted against maintaining the accelerated approval of pembrolizumab [28,29,30,31], which led to subsequent withdrawal of this drug for use in this setting. Regarding MSI-high tumors, the study published by Le et al. led to an unprecedented approval of pembrolizumab for MSI-high solid tumors including GECs [32]. Monotherapy with pembrolizumab can result in long-lasting control of metastatic GECs that are MSI-high, although MSI-high tumors only constitute about 2–3% of metastatic GECs [33]. Moreover, emerging evidence from peri-operative studies support use of ICIs among patients with esophageal/GEJ AC or SCC that have had CRT followed by surgery with R0 resection, with non-pathologic complete response (CR) who are otherwise eligible for immunotherapy, particularly in PD-L1 CPS ≥ 5 tumors. In this article, we will review the results of studies that have evaluated the utility of ICI in the third-line (or higher), second-line, first-line, and peri-operative treatment settings of GECs. Of note, we will review the current standards according to the American guidelines and approvals, while the EMA guidelines are not reflected in this paper; however in brief, EMA has approved pembrolizumab for use in esoSCC and GEJ AC in CPS ≥ 10 tumors, as well as nivolumab for gastro-esophageal AC in CPS ≥ 5 tumors.

Table 1 summarizes the results of the discussed trials that evaluated the safety and efficacy of ICI for the treatment of GEC in different settings.

## 2. The Use of ICI in the Third-Line Setting

The utility of ICI in the third-line treatment of GECs has been previously studied in multiple phase 2 and phase 3 trials [28,34,35]. Among the third-line studies, cohort 1 of the KEYNOTE-059 enrolled patients who were started on pembrolizumab 200 mg every three weeks as monotherapy until disease progression, investigator, or patient decision to withdraw, or unacceptable toxic effects [28]. With a median response duration of 8.4 months, the overall objective response rate (ORR) was 11.6%, with CR in 2.3% of patients. PD-L1 positivity was defined as CPS ≥ 1 (22C3 assay), and ORR was higher in this population, reported as 15.5% of PD-L1 positive patients, with a longer median response duration of 16.3 months among these patients. However, after excluding MSI-high tumors, the ORR was 13.3% in the remaining microsatellite stable (MSS) PD-L1 CPS ≥ 1 tumor. In September 2017, pembrolizumab received a conditional approval for third-line or higher treatment of advanced/recurrent GEC in tumors with PD-L1 CPS ≥ 1 according to the results of KEYNOTE-059; however, in April 2021, FDA’s ODAC voted against maintaining the conditional approval of pembrolizumab in this setting. This decision was made based on the updated results of phase 3 KEYNOTE-061 and KEYNOTE-062 trials that investigated pembrolizumab monotherapy in gastric/GEJ cancers in the second-line and monotherapy and in combination with chemotherapy in the first-line, respectively [36,40]. In KEYNOTE-061, monotherapy with pembrolizumab compared paclitaxel failed to meet its primary end point of OS (HR, 0.82; 95% CI, 0.66–1.03; *p* = 0.042). Additionally, the KEYNOTE-062 trial investigated pembrolizumab both as monotherapy and in combination with cisplatin and fluoropyrimidine chemotherapy; neither pembrolizumab monotherapy nor the combination therapy were superior for OS. This led to subsequent withdrawal of pembrolizumab for third-line or higher line treatment of advanced PD-L1 CPS ≥ 1 GEC in July 2021.

Other immune-checkpoint inhibitors such as nivolumab and avelumab have also been studied in the third-line settings. In ATTRACTION-02, patients in Asia with unresectable advanced or recurrent gastric/GEJ cancer after failure of two or more previous chemotherapy regimens were randomized in a 2:1 ratio to receive nivolumab versus placebo [34,53]. The investigators reported a mOS of 5.32 months versus 4.14 months in the nivolumab and placebo groups, respectively [34]. Therefore, this trial demonstrated a survival benefit with nivolumab use which led to its approval for use in the third-line setting in Asia but not in the United States [54]. While PD-L1 by TPS (28-8 assay) did not appear to be helpful in predicting whether patients would derive benefit, CPS scoring (CPS ≥ 1) in the limited samples available for this was predictive [55]. Another notable study is JAVELIN-300, in which patients with unresectable, recurrent, locally advanced, or metastatic gastric/GEJ cancer were randomized to either avelumab versus physician’s choice of chemotherapy (paclitaxel or irinotecan, while patients (*n* = 3) who were ineligible for chemotherapy received best supportive care) [35]. The trial did not meet its primary and secondary end points and in fact appeared worse for OS, progression-free survival (PFS), and ORR in the avelumab versus chemotherapy arms, respectively [35]. Notably, JAVELIN-300 was the only trial with a randomized design with a truly relevant standard control arm in the third-line setting. PD-L1 expression of subjects was reported using the TPS, which was not predictive of response to treatment with avelumab.

In conclusion, the totality of the data in the third-line setting do not support the utility of anti-PD-1/L1 monotherapy compared to active therapy such as standard chemotherapy. However, monotherapy appears to be beneficial in patients with MSI-high tumors or potentially for patients with very high PD-L1 expression (CPS ≥ 10), either of which who have not previously received ICIs for whatever reason.

## 3. The Use of ICI in the Second-Line Setting

ICIs have also been evaluated for second-line treatment of GECs: KEYNOTE-061, was a randomized, open-label, phase 3 study that evaluated pembrolizumab versus paclitaxel for second-line treatment of advanced gastric or GEJ cancer, irrespective of PD-L1 status [36]. Patients were randomized to receive either pembrolizumab 200 mg every 3 weeks for up to 2 years or standard-dose paclitaxel. Enrollment to the study for PD-L1 negative tumors was terminated during the conduct of the study. For PD-L1 CPS ≥ 1 tumor (22C3 assay), pembrolizumab and paclitaxel groups had mOS of 9.1 months (95% CI 6.2–10.7) and 8.3 months (95% CI 7.6–9.0), respectively (HR 0.82, 95% CI 0.66–1.03; one-sided *p* = 0.0421). For PD-L1 CPS ≥ 1 tumor, the median PFS in pembrolizumab and paclitaxel groups were 1.5 months (95% CI 1.4–2.0) and 4.1 months (95% CI 3.1–4.2), respectively (HR 1.27, 95% CI 1.03–1.57). According to these results, second-line therapy with pembrolizumab did not significantly improve OS over paclitaxel for advanced gastric or GEJ cancer with PD-L1 CPS ≥ 1 [36]. In fact, there was crossing of the curves, suggesting even after enriching with CPS ≥ 1 PD-L1 tumors, there was still a group of patients that was having worse outcomes compared to chemotherapy, and others having better outcomes. In particular, there did appear to be benefit in patients with CPS ≥ 10 as well as MSI-high tumors in an unplanned subgroup analysis (evaluation of CPS ≥ 10 was a post-hoc analysis and not a prespecified cohort). Notably, in the 200 randomized patients with PD-L1 CPS negative tumors, the mOS was ~5 months versus ~8 months and mPFS ~1 month versus ~4.5 months in the pembrolizumab and paclitaxel arms, respectively, showing clear inferiority of pembrolizumab to what might be considered substandard second-line therapy with paclitaxel alone (given that the RAINBOW study demonstrated superiority of paclitaxel and ramucirumab compared to paclitaxel alone) [56].

In KEYNOTE-181, patients with advanced or metastatic SCC or AC of the esophagus that had progressed after first-line therapy were randomized to receive either pembrolizumab or investigator’s choice of chemotherapy (paclitaxel, docetaxel, or irinotecan) irrespective of their PD-L1 status [37]. In this study, patients with one of the co-primary endpoints, CPS ≥ 10 had longer OS in the pembrolizumab group versus chemotherapy (22C3 assay; mOS 9.3 vs. 6.7 months; HR 0.69, 95% CI 0.52–0.93; *p* = 0.0074). Also, median OS was 8.2 months versus 7.1 months (HR, 0.78 (95% CI, 0.63–0.96); *p* = 0.0095) in patients with co-primary endpoint of all SCC, but was not different in all patients enrolled (all histology and any PD-L1) with 7.1 months versus 7.1 months (HR, 0.89 (95% CI, 0.75 to 1.05); *p* = 0.0560). Subgroup analyses demonstrated that there was no significant benefit in patients with CPS ≤ 10 tumors of either histology. Therefore, although pembrolizumab therapy resulted in a significantly longer OS in patients with CPS ≥ 10 (any histology) or those with SCC (any PD-L1 status), such difference was not observed among all comers in the intention to treat (ITT) analysis nor in CPS < 10 (any histology). The results were also equivocal with small numbers in the CPS ≥ 10 adenocarcinoma subgroup. This led to the FDA approval (7/30/2019) of pembrolizumab as second-line treatment only of patients with esophageal SCC with PD-L1 CPS ≥ 10.

Similarly, ATTRACTION-3 included patients with unresectable advanced or recurrent esophageal SCC (regardless of PD-L1 expression) refractory or intolerant to one previous fluoropyrimidine-based and platinum-based chemotherapy [38]. Patients were randomly assigned to either nivolumab or the investigator’s choice of chemotherapy (paclitaxel or docetaxel). Median follow-up for OS in the nivolumab and chemotherapy groups were 10.5 months (IQR 4.5–19.0) and 8.0 months (4.6–15.2), respectively. Moreover, OS was significantly improved in the nivolumab group versus chemotherapy group (median 10.9 months, 95% CI 9.2–13·3 vs. 8.4 months, 7.2–9.9; HR 0.77, 95% CI 0.62–0·96; *p* = 0.019, after a minimum follow up of 17.6 months) [38]. It is notable that in ATTRACTION-3, about 50% and 30% of all comers had TPS ≥ 1% and ≥ 10%, respectively. Higher TPS scores trended to better outcomes (e.g., TPS ≥ 10% vs. < 10% HR 0.69 vs. HR 0.8, respectively). CPS scoring information was not provided. It is interesting to note that even in this study with only SCC tumors, that there was some crossing of the survival curves as was seen in KEYNOTE-061, particularly in PFS but also slightly in OS. Results of ATTRACTION-3 were in line with results of KEYNOTE-181, where both studies showed benefit for ICI in the second-line treatment of esoSCC, particularly with high PD-L1 expression. However, nivolumab was approved for esoSCC for second-line therapy in the United States irrespective of PD-L1 status.

Another notable study in the second-line setting is RATIONALE 302, which was recently presented at ASCO 2021 [39]. In this global, phase 3 study, patients with esoSCC who had advanced/unresectable or metastatic disease and progression after first-line systemic chemotherapy were randomized to receive either tislelizumab (200 mg IV every 3 weeks) or investigator-chosen chemotherapy. Overall, 512 patients (79% Asian) were recruited from 10 countries. Out of the 256 patients in the tislelizumab group, 157 patients (61%) had CPS ≥ 10. After a median follow up of 8.5 months of the tislelizumab group and 5.8 months of the chemotherapy group, treatment with tislelizumab demonstrated an improvement in OS in the ITT population (mOS 8.6 vs. 6.3 month; HR 0.70, 95% CI 0.57–0.85; *p* < 0.001). The benefit was most pronounced in the PD-L1 CPS ≥ 10 population (mOS 10.3 vs. 6.8 month; HR 0.54, 95% CI: 0.36–0.79, *p* = 0.0006). Results for CPS < 10 tumors were not shown. Survival benefit was consistently observed across pre-defined subgroups and regions [39].

## 4. The Use of ICI in the First-Line Maintenance Setting

JAVELIN Gastric 100 study evaluated the use of avelumab as maintenance after first-line induction chemotherapy in patients with gastric cancer irrespective of PD-L1 status [52]. JAVELIN Gastric 100 was a global, open-label, phase 3 trial which included patients with untreated/unresectable, HER2-negative, any PD-L1, locally advanced or metastatic gastric or GEJ AC. Of 805 patients enrolled to induction chemotherapy, 499 (62%) were stable and otherwise eligible and randomly assigned to maintenance treatment. Patients who did not have progression of disease after 12 weeks of first-line chemotherapy were randomly assigned to avelumab every 2 weeks or continued chemotherapy. In the ITT group, mOS was 10.4 months (95% CI 9.1–12.0) with avelumab versus 10.9 months (95% CI 9.6–12.4) with chemotherapy. Furthermore, the 24-month OS rate was 22.1% with avelumab versus 15.5% with chemotherapy (HR 0.91; 95% CI, 0.74–1.11; *p* = 0.1779). However, it is notable that despite the 38% drop off of quick progressing disease after only 3 months of therapy among patients enrolled to induction, there was still an observed crossing of the curves from the time of randomization, with a group of patients doing worse with avelumab compared to continued chemotherapy, and a group doing better. In the PD-L1-positive subgroup (TPS ≥ 1%, 73-10 assay, *n* = 54), the HR for OS was 1.13 (95% CI 0.57–2.23; *p* = 0.6352); however, in patients with CPS ≥ 1 tumors (22C3 assay, *n* = 137), mOS was 14.9 months (95% CI 8.7–17.3) and 11.6 months (95% CI 8.4–12.6) with avelumab and chemotherapy, respectively (unstratified HR 0.72; 95% CI 0.49–1.05). Accordingly, this study did not show an OS benefit with avelumab maintenance versus continued chemotherapy in patients with advanced gastric or GEJ cancer among all patients or those with PD-L1 TPS positive tumors. However, there was a signal consistent with previous studies suggesting CPS scoring as the best enriching biomarker [52].

## 5. The Use of ICI in the First-Line Setting

The first notable study in this group is KEYNOTE-062, which was a global, randomized placebo-controlled three-arm phase 3 trial evaluating pembrolizumab monotherapy open-label vs. pembrolizumab in combination with chemotherapy (either cisplatin/5-FU or cisplatin/capecitabine) vs. chemotherapy plus placebo in the first-line setting for only patients selected to have tumors PD-L1 CPS ≥ 1 22C3 assay [40]. In the monotherapy ICI versus chemotherapy analysis, although at median follow-up of 29.4 months pembrolizumab was stated to be non-inferior to chemotherapy for OS in patients with CPS ≥ 1 (median, 10.6 vs. 11.1 months; HR 0.91; 99.2% CI, 0.69–1.18), it was also demonstrated that pembrolizumab monotherapy was not superior to chemotherapy, and in fact as was seen in every previous study with appropriate comparator arms, there were two populations of patients having clearly discrepant outcomes. The curves were observed to be crossing much like demonstrated in JAVELIN-100, KEYNOTE-061, and also the other studies that showed this explicitly or implicitly. In a preplanned analysis, pembrolizumab prolonged OS versus chemotherapy in patients with CPS ≥ 10 (median, 17.4 vs. 10.8 months; HR 0.69; 95% CI, 0.49–0.97), but this difference was not statistically tested due to predefined rules (CPS ≥ 10 was an amended co-primary endpoint); additionally, these curves yet again also crossed suggesting that this PD-L1 criterion was not enough to completely enrich for those likely to benefit and limit detriment from pembrolizumab monotherapy compared to chemotherapy, in contrast to what was observed with the same threshold used in KEYNOTE-061. In the therapeutic combination analysis, pembrolizumab plus chemotherapy was not superior to placebo plus chemotherapy for OS in patients with CPS ≥ 1 (12.5 vs. 11.1 months; HR 0.85; 95% CI 0.70–1.03; *p* = 0.05) or CPS ≥ 10 (12.3 vs. 10.8 months; HR 0.85; 95% CI 0.62–1.17; *p* = 0.16) or for PFS in patients with CPS ≥ 1 (6.9 vs. 6.4 months; HR 0.84; 95% CI 0.70–1.02; *p* = 0.04). In fact, the Kaplan-Meier OS curves (pembrolizumab vs. chemotherapy) are notable for an early but slight favorable trend toward chemotherapy. The violation of the proportional hazard assumption in studies such as KEYNOTE-062 and the others suggests that while a group of patients benefited more from ICI, some patients did worse (even in CPS ≥ 10). Meanwhile, there was a sustained separation of curves in favor of pembrolizumab after 12 months. This observation indicates a long-term survival benefit with pembrolizumab in a subgroup of patients (2 year OS rates of 27% vs. 19%), particularly MSI-high tumors [40].

ATTRACTION-04 phase 3 study evaluated the efficacy and safety of nivolumab in combination with chemotherapy in unresectable advanced or recurrent gastric/GEJ cancer irrespective of PD-L1 status, and compared nivolumab to placebo in combination with S-1/oxaliplatin (SOX) or capecitabine/oxaliplatin (CAPOX) chemotherapy [41]. The co-primary endpoints were PFS and OS. Patients were recruited from Japan, Taiwan, and Korea, and stratified by PD-L1 positivity, defined as TPS ≥ 1 28-8 assay. Only 16% of patients had PD-L1 TPS ≥ 1%. Each arm of the study included 362 patients, out of which 229/230 and 130/128 received SOX and CAPOX in nivolumab/placebo arms, respectively. Median PFS was 10.4 month (95% CI 8.44–14.75) and 8.3 months (95% CI 6.9–9.4) in the nivolumab + chemo and placebo + chemo groups, respectively (HR 0.68; 98.5% CI 0.51–0.90; *p* = 0.0007). The one-year PFS rate was 45.4% and 30.6% in the nivolumab + chemo and placebo + chemo arms, respectively. However, the analysis of OS did not show a significant difference between the two arms: the mOS was 17.4 (95% CI 15.67–20.83) and 17.1 (95% CI 15.18–19.65) months in the nivolumab + chemo and placebo + chemo arms, respectively (HR 0.90, 95% CI 0.75–1.08; *p* = 0.25). Moreover, 27% of the patients in the control arm received ICI in the second-line of their treatment, which essentially would not be enough to affect the median survival rate of the control arm. Notably, the mOS in the placebo + chemo group was 17.1 months, which is longer than the mOS reported in other studies such as CHECKMATE-649. The reason for such a longer OS of the control arm of this study remains unclear, however the Asian patient population, patient selection, and post-progression therapy differences are considered as the contributing causes for this observation, which has been seen in several other studies. The incidence of PD-L1 high versus low, and the outcomes by PD-L1 CPS have not been reported for ATTRACTION-04, yet CPS high versus low scoring would likely result in a significant difference in outcome between the two arms, as has been observed in all studies that have reported such outcomes to date. It is possible that ATTRACTION-04 was negative for OS due to lower incidence of higher PD-L1 tumors enrolled; evaluation and presentation of the incidence/outcomes of this biomarker within this study would therefore be important.

The randomized, multicenter, three-arm phase 3 trial that evaluated ICI with nivolumab for first-line use of previously untreated advanced adenocarcinomas of the esophageal, GEJ and stomach irrespective of PD-L1 status is CHECKMATE-649 [42]. This study was initially designed to compare OS in the two arms of patients randomized to nivolumab + ipilimumab open label versus chemotherapy alone (oxaliplatin + fluoropyrimidine, CAPOX or FOLFOX). Several changes were made to the study design since enrollment initiation, including the addition of a third arm of open label nivolumab + chemotherapy (CAPOX or FOLFOX) arm and the discontinuation of the nivolumab + ipilimumab arm after ~450 patients were enrolled to it. Stratification factors included PD-L1 TPS ≥ 1% or < 1% (28-8 assay). Regarding the nivolumab + ipilimumab arm, a recent press release reported that OS was not improved in the PD-L1 CPS ≥ 5 compared to chemotherapy alone [57], and report at ESMO 2021 revealed that nivolumab + ipilimumab was not superior to chemotherapy, and the OS curves again showed crossing of the curves classic for chemo-free immunotherapy approaches seen previously, even in the CPS ≥ 5 subgroup [58]; the median PFS was significantly worse with 2.8 months in the nivolumab + ipilimumab arm versus 6.3 months in the chemotherapy arm. Regarding the chemotherapy with/without nivolumab comparison, in the largest study conducted to date for this disease, a total of 1581 patients irrespective of PD-L1 status were randomized to the nivolumab + chemotherapy (789) and chemotherapy only (792) arms. Dual primary endpoints were OS and PFS (defined in the sub-population with PD-L1 CPS ≥ 5). Among enrolled participants, 24.7% of patients were Asian, and 82% had a tumor PD-L1 CPS ≥ 1, and 60.4% with CPS ≥ 5. Among patients with CPS ≥ 5, the incidence of TPS ≥ 1% was 24.1%. With a minimum follow up of 12.1 months, the mOS was significantly different between the two arms of patients with CPS ≥ 5: 14.1 months (95% CI 13.1–16.2 months) and 11.1 months (95% CI 10.0–12.1 months) in the nivolumab + chemotherapy and chemotherapy only groups, respectively (HR 0.71, 98.4% CI 0.59–0.86, *p* < 0.001). The 12-month OS rate was 57% and 46% in the nivolumab + chemotherapy and chemotherapy only groups, respectively; although this analysis was only done among patients whose tumors expressed PD-L1 CPS ≥ 5. Any treatment related adverse event that were grade 3 or higher were 15% higher in the nivolumab + chemotherapy versus chemotherapy alone arm, and serious treatment related adverse events grade 3 or higher were 7% higher in the nivolumab + chemotherapy versus chemotherapy alone arm. With respect to secondary outcomes, there was a superior OS benefit with nivolumab + chemotherapy in PD-L1 CPS ≥ 1 (mOS of 14.0 months with 95% CI 12.6–15 in nivolumab + chemotherapy vs. mOS of 11.3 months with 95% CI 10.6–12.3 in the chemotherapy only group; HR 0.77, 99.3% CI 0.64–0.92, *p* < 0.001) as well as among all randomized patients regardless of their PD-L1 CPS (mOS of 13.8 months with 95% CI 12.6–14.6 in nivolumab + chemotherapy vs. mOS of 11.6 months with 95% CI 10.9–12.5 in the chemotherapy only group; HR 0.80, 99.3% CI 0.68–0.94, *p* = 0.0002). However, as the numbers imply, the extent of benefit with nivolumab + chemotherapy in secondary endpoints was diluted. As anticipated, data were eventually revealed demonstrating that benefit was solely among patients with PD-L1 CPS ≥ 5, with OS HR 0.92 (mOS 13.1 vs. 12.5 in CPS < 1) and HR 0.94 (mOS 12.4 vs. 12.3 in CPS < 5), and similarly non-significant differences for PFS and ORR among these low/negative PD-L1 by CPS subgroups. Despite this, FDA approved nivolumab for all patients with gastroesophageal AC irrespective of PD-L1 score (16 April 2021). However, similar to KEYNOTE-590 below, the recent NCCN guidelines have listed nivolumab with oxaliplatin as category 1 for tumors with PD-L1 CPS ≥ 5. Tumors with PD-L1 CPS 1-4 have category 2B recommendation, and PD-L1 CPS 0 is currently not recommended.

ORIENT-16 was a randomized, double-blind, phase 3 trial that evaluated the efficacy and safety of sintilimab plus CAPOX vs. placebo plus CAPOX for first-line treatment of advanced gastric and GEJ AC among Chinese patients [45]. In the prespecified interim analysis, 650 patients were randomized to receive sintilimab plus CAPOX (*n* = 327) and placebo plus CAPOX (*n* = 323). The addition of sintilimab to CAPOX showed superior OS benefit in patients with CPS ≥ 5 tumors (median 18.4 vs. 12.9 mo; HR 0.66; 95% CI 0.50–0.86; *p* < 0.002) and among all patients (median 15.2 vs. 12.3 mo; HR 0.76; 95% CI 0.62–0.93; *p* < 0.009). For patients with CPS ≥ 10 tumors, the median OS was 20.0 months versus 11.3 months, in favor of the sintilimab arm. Although these results are encouraging, the data regarding patients with lower CPS levels was not explicitly reported, yet the results do show the same phenomenon as seen with CHECKMATE-649, with less pronounced HRs as patients with lower PD-L1 levels were added to the analyses [45]. These results are consistent with prior studies and support the use of sintilimab in patients with CPS ≥ 10 tumors.

The results of KEYNOTE-590, which is a randomized phase 3 trial comparing pembrolizumab + cisplatin/5-FU vs. placebo + cisplatin/5-FU in the first-line setting irrespective of PD-L1 status, were presented initially at the ESMO 2020 virtual conference [46]. Patients with locally advanced/unresectable or metastatic AC, esoSCC, or Siewert type 1 GEJ AC were enrolled and randomized to pembrolizumab or placebo every three weeks for up to two years plus chemotherapy (cisplatin + 5-FU) [46]. Treatment continued until progression, unacceptable toxicity, or withdrawal, or for up to two years. The median follow-up of 749 patients was 10.8 months. In this trial, pembrolizumab plus chemotherapy vs. chemotherapy was superior for OS in patients in co-primary endpoints with esoSCC with CPS ≥ 10 22C3 assay (median 13.9 vs. 8.8 month; HR 0.57; 95% CI 0.43–0.75; *p* < 0.0001). The OS benefit was also seen in patients with esoSCC irrespective of PD-L1 (median 12.6 vs. 9.8 months; HR 0.72; 95% CI 0.60–0.88; *p* = 0.0006). The OS benefit was observed among patients who had any histology disease with high PD-L1 expression define as CPS ≥ 10 (median 13.5 vs. 9.4 months; HR 0.62; 95% CI, 0.49–0.78; *p* < 0.0001), as well as among all patients (median 12.4 vs. 9.8 months; HR, 0.73, 95% CI 0.62–0.86; *p* < 0.0001). However, CPS < 10 tumors clearly did not derive any significant OS benefit (HR, 0.86, 95% CI 0.68–1.10), including non-statistically significant difference in both histologies SCC and AC with the CPS < 10 cohort. PFS was superior with pembrolizumab plus chemotherapy vs. chemotherapy in esoSCC (median 6.3 vs. 5.8 month; HR 0.65; 95% CI 0.54–0.78; *p* < 0.0001). Superiority of PFS in pembrolizumab group was also observed in patients with CPS ≥ 10 tumors (median 7.5 vs. 5.5 months; HR 0.51; 95% CI 0.41–0.65; *p* < 0.0001), as well as in all patients (median 6.3 vs. 5.8 months; HR 0.65; 95% CI 0.55–0.76; *p* < 0.0001). The treatment related adverse events that were grade 3 or higher were 5% higher in the pembrolizumab arm compared to placebo, and immune-mediated adverse events and infusion reactions of any grade were 14% higher in the pembrolizumab arm. In this study, 52.5% of the patients were Asian, 73.5% had SCC, and 49.9% had PD-L1 CPS ≥ 10. Considering such patient demographic and relatively high rate of high PD-L1 CPS ≥ 10 expressions compared to other previous studies including KEYNOTE-062, the KEYNOTE-590 trial was enriched for patients likely to benefit from pembrolizumab, likely resulting in the observed OS benefit in the ITT analysis. Therefore, while the FDA approved pembrolizumab for first-line therapy for esophageal/GEJ cancers irrespective of histology and PD-L1 (March 2021), the EMA approved pembrolizumab only for tumors with PD-L1 CPS ≥ 10 in June 2021. In addition, similar to the tiered recommendation of nivolumab from CHECKMATE-649, the NCCN guidelines have listed first-line pembrolizumab with chemotherapy (either cisplatin category 1 or oxaliplatin category 2A) for tumors with PD-L1 CPS ≥ 10 for both SCC and AC esophageal/GEJ cancers, while tumors with PD-L1 CPS 1-9 have category 2B recommendation, and PD-L1 CPS 0 is currently not recommended [59].

Another notable study that evaluated the efficacy of ICI for first-line treatment of advanced esoSCC is CHECKMATE-648 [47]. In this global phase 3 study, patients with untreated, unresectable advanced, recurrent, or metastatic esoSCC were enrolled regardless of their PD-L1 expression. Patients were randomized to receive nivolumab (240 mg q 2 weekly) + chemotherapy (5-FU + cisplatin), nivolumab (3 mg/kg q 2 weekly) + ipilimumab (1 mg/kg q 6 weekly), or chemotherapy alone. Among the 970 patients, 49% had TPS ≥ 1%. Improvement in OS were observed in the nivolumab + chemotherapy and nivolumab + ipilimumab compared to patients in the chemotherapy alone group: among patients with TPS ≥ 1% 28-8 assay, mOS of those in nivolumab + chemotherapy, nivolumab + ipilimumab and chemotherapy alone groups were 15.4 months (95% CI 11.9–19.5; HR vs. chemo alone: 0.54, 99.5% CI 0.37–0.8; *p* < 0.001), 13.7 months (95% CI 11.2- 17.0; HR vs. chemo alone: 0.64, 98.6% CI 0.46–0.90; *p* = 0.001) and 9.1 months (95% CI 7.7–10.0), respectively. Among all randomized patients, mOS of those in nivolumab + chemotherapy, nivolumab + ipilimumab and chemotherapy alone groups were 13.2 months (95% CI 11.1–15.7; HR vs. chemo alone: 0.74, 99.1% CI 0.58–0.96; *p* = 0.002), 12.8 months (95% CI 11.3- 15.5; HR vs. chemo alone: 0.78, 98.2% CI 0.62–0.98; *p* = 0.011) and 10.7 months (95% CI 9.4–11.9), respectively. Additionally, there was no significant OS difference between the arms of the study in the TPS < 1% group (HR 0.98, not significant). Statistically significant PFS benefit was also observed for nivolumab + chemo vs. chemo (HR 0.65, 98.5% CI 0.46–0.92; *p* = 0.0023) in patients with TPS ≥ 1%; however, PFS in nivolumab + ipilimumab vs. chemo did not meet the prespecified boundary for significance even among patients with TPS ≥ 1%, and in fact, in line with all previous chemo-free approaches, both of the OS and PFS curves evaluating nivolumab + ipilimumab versus chemotherapy crossed. Any treatment related adverse event that was grade 3 or higher was 11% higher in the nivolumab + chemotherapy arm versus the chemotherapy alone arm. While nivolumab + chemotherapy demonstrated superior OS vs. chemotherapy alone along with durable objective responses and acceptable safety in patients with advanced esoSCC, nivolumab + ipilimumab demonstrated an absolutely lower mOS compared to nivolumab + chemotherapy (13.7 versus 15.4 months) and demonstrated the familiar crossing of the curves, along with a 10% higher grade 3 or higher serious treatment related adverse events compared to chemotherapy and 5% higher compared to nivolumab + chemotherapy [47].

The results of the phase 3 ESCORT-1st study which evaluated the efficacy and safety of camrelizumab plus chemotherapy vs. chemotherapy in Asian patients with untreated advanced or metastatic esoSCC irrespective of PD-L1 status were recently presented [48]. A total of 596 patients were randomized to receive either camrelizumab or placebo, both in combination with paclitaxel and cisplatin for up to 6 cycles. PD-L1 expression was assessed at a central laboratory (Shuwen Biotech, Deqing, Zhejiang, China) while using the 6E8 antibody, Abcam, as the PD-L1 immunohistochemistry kit. The incidence of PD-L1 TPS > 1% was 55.2%, >5% was 47.8%, and >10% was 33.9%. With a median follow-up of 10.8 months, camrelizumab plus chemotherapy showed a superior OS benefit (median 15.3 month; 95% CI 12.8–17.3 vs. 12.0 months; 95% CI 11.0–13.3; HR 0.70, 95% CI 0.56–0.88; *p* = 0.001). Camrelizumab plus chemotherapy was also superior for PFS vs. placebo plus chemotherapy (median 6.9 months; 95% CI 5.8–7.4 vs. 5.6 months; 95% CI 5.5–5.7; HR 0.56, 95% CI 0.46–0.68; *p* < 0.0001) [48]. However, the OS differences in each of the PD-L1 < 10%, <5% and <1% subgroups were not statistically significant. It is likely, as has been observed in all previous studies, that CPS analysis would further distinguish patients who do and do not derive benefit from anti-PD1/PD-L1 therapies. Results of this study showed that addition of camrelizumab to chemotherapy may improve PFS and OS with a manageable safety profile, suggesting a potential role in the first-line treatment of patients with advanced or metastatic esoSCC, particularly with high expressing PD-L1.

Another notable study that evaluated the efficacy and safety of sintilimab plus chemotherapy versus chemotherapy as first-line treatment in Asian patients (about 97%) with unresectable locally advanced, recurrent or metastatic esoSCC is ORIENT-15 [49]. A total of 659 patients were randomized to receive sintilimab (*n* = 327) versus placebo (*n* = 332) plus chemotherapy with cisplatin and 5FU or cisplatin and paclitaxel. In the interim analysis of the study and with a median follow up of 11.4 months, sintilimab plus chemo showed superior OS benefit in all patients (median 16.7 vs. 12.5 mo, HR 0.62; 95% CI 0.508–0.777, *p* < 0.0001) as well as among patients with CPS ≥ 10 (median 17.2 vs. 13.6 mo, HR 0.63; 95% CI 0.48–0.84, *p* < 0.0001). Also, PFS was superior with sintilimab plus chemo in all patients (median 7.2 vs. 5.7 mo, HR 0.55; 95% CI 0.46–0.67, *p* < 0.0001) and in patients with CPS ≥ 10 (median 8.3 vs. 6.4 mo, HR 0.58; 95% CI 0.449–0.749, *p* < 0.0001). In contrast to KEYNOTE-590, ESCORT-1 and CHECKMATE-648 SCC studies, benefit was observed across all CPS groups including lower PD-L1 expressing tumors. The promising results of this study also demonstrated another potential role for sintilimab for first-line treatment of advanced or metastatic esoSCC [49].

The safety and efficacy of toripalimab for first-line treatment of advanced or metastatic esoSCC has been shown in the JUPITER-06 interim analysis report [50]. In this study that was conducted among the Asian population, patients were randomized to receive toripalimab (*n* = 257) or placebo (*n* = 257) in combination with paclitaxel and cisplatin up to 6 cycles, followed by toripalimab or placebo maintenance. With median follow-up of 7.4 and 7.3 months in the two arms, there was a significant improvement in OS for toripalimab over placebo (HR 0.58; 95% CI 0.43–0.78; *p* < 0.00037) with median OS of 17.0 vs. 11.0 months [50]. As seen with ORIENT-15, benefit was seen across all CPS groups. Thus, there is discrepancy between SCC studies with respect to PD-L1 as a predictive biomarker for unclear reasons and warrants further studies to explain these differences. From JUPITER-06, toripalimab may serve as yet another potential option for patients with advanced or metastatic esoSCC [50].

Emerging evidence suggests that combination strategies can be used to improve immune engagement and augment response to immune checkpoint inhibition [16,18,57]. In fact, using ICI in combination with anti-HER2 in HER2 amplified tumors has been previously investigated and demonstrated potential synergy [16,18]. The combination of margetuximab, an Fc engineered monoclonal HER2 antibody, with pembrolizumab as a chemo-free approach demonstrated safety and efficacy as second-line therapy, particularly pronounced in HER2 IHC3+ and PD-L1 CPS ≥ 1 patients [16], with the response rates observed with this combination being far higher than expected for either agent alone. This could be attributed to the synergistic activity between the innate (antibody-dependent cellular toxicity) and adaptive (CD8-mediated) immune responses. The ongoing global, randomized, double-blind, placebo-controlled phase 3 KEYNOTE-811 study is assessing whether adding pembrolizumab to standard of care improves efficacy among patients with HER2+ metastatic gastric/GEJ cancer irrespective of PD-L1 status [51]. In this study, patients with previously untreated, unresectable, or metastatic HER2-positive gastric/GEJ cancer are randomized to pembrolizumab 200 mg q 3 weekly or placebo. All patients receive trastuzumab and investigator’s choice of 5-FU and cisplatin (FP) or CAPOX. Among the first 264 patients enrolled, interim data have been recently reported with a confirmed ORR (95% CI) of 74.4% (66.2–81.6) for pembrolizumab + standard of care versus 51.9% (43.0–60.7) for placebo + standard of care (difference, 22.7 percentage points [95% CI, 11.2–33.7], *p* = 0.00006); CR rate was 11.3% vs. 3.1% and DCR (95% CI) was 96.2% (91.4–98.8) vs. 89.3 (82.7–94.0). The incidence of PD-L1 CPS > 1 22C3 assay was 86% (228/264), while the incidence of CPS > 5 or > 10 are not yet reported; similarly outcomes by PD-L1 by these various cut-offs have not yet been reported. [51]. The results of this interim analysis reporting ORR from the phase II portion of the study led to FDA approval (5/5/2021) of this strategy in the first-line setting for clinically HER2-positive gastric/GEJ AC.

Another notable ongoing trial among patients with HER2-positive GEC is the MAHOGANY study which is a 2-cohort study in unresectable metastatic/locally advanced GEC. In cohort A, the safety and efficacy of margetuximab plus retifanlimab was evaluated in biomarker selected population of HER2 IHC3+, PD-L1 CPS ≥ 1 and non-MSI-high patients. In cohort A, a total of 43 patients (23 with gastric, 18 with GEJ AC; 36 with metastatic disease) were enrolled. Tumor shrinkage was seen in 32 (78%) of 40 evaluable patients, with at least one post base-line target lesion measurement. The ORR was 21/40 (52.5%) and the median duration of response was 10.3 months (95% CI 4.57-NE). The disease control rate was 29/40 (72.5%). The mPFS, 6 months PFS rate, 9 months PFS rate and 12 months PFS rate were 6.4 (95% CI 6.01-NE), 71% (95% CI 53–83), 50% (95% CI 31–66), and 50% (95% CI 31–66), respectively. In terms of OS, both 12 months and 18 months OS rates were 85% (95% CI 63–95). Most common treatment related adverse effect (TRAE) was fatigue (21%), followed by infusion-related reaction (19%), rash (19%), diarrhea (16%), and pruritis (16%). The overall TRAE occurred in 18.6% (8/43) of patients. In contrast to other previous chemotherapy-free approaches in this disease with targeted or ICI, there was not a steep decline in PFS or OS with this approach. For example, in KEYNOTE-062 monotherapy pembrolizumab in PD-L1 CPS > 1 tumor had a mPFS of only 2 months, and ORR of only 14.8% (38/250). Moreover, in the phase 2 study evaluating trastuzumab/pembrolizumab and chemotherapy [18], patients were allowed to have one cycle of trastuzumab/pembrolizumab without chemotherapy and restaging after albeit only one cycle demonstrated only a 9% ORR, potentially emphasizing better efficacy of margetuximab versus trastuzumab. Thus, the results from MAHOGANY cohort A part 1 suggested that the combination of margetuximab plus retifanlimab is well tolerated, and with strict biomarker selection this regimen may provide for an effective and safe chemo-free approach for newly diagnosed patients with HER2 IHC3+/PD-L1 CPS ≥ 1 positive GEC as a first pass treatment, with the advantage of delaying therapy to only when/if there is disease progression. In fact, with more than half of patients on therapy for more than 10 months, and many continuing to date for almost 2 years, toxicities from the chemotherapy can be delayed or in some cases avoided altogether in extreme responders.

The updated results of the phase Ib/II PANTHERA trial which evaluated pembrolizumab, trastuzumab, and chemotherapy as first-line therapy for HER2-positive advanced gastric and GEJ cancers were reported in ASCO 2021 [60]. Among total of 38 patients, 55.3% had CPS ≥ 1 and 13.2% had CPS ≥ 10. ORR was 76.7% (CR 16.3%, PR 60.5%, conversion surgery 4.6%). The median PFS and median OS were 8.6 months (95% CI 7.2–16.5) and 19.3 months (95% CI 16.5-NR), respectively. The results of this study are in line with the previous reports, that combination HER2-directed therapy and ICI may have promising efficacy based on HER2 amplification by NGS in advanced gastric/GEJ AC [60].

## 6. The Use of ICI in the Peri-Operative/Adjuvant Setting

Treatment of locally advanced GEC that is amenable to surgery involves either a combined-modality neoadjuvant CRT or chemotherapy peri-operatively [4]. The two notable trials in this regard include the CROSS and FLOT4 trials: In the CROSS trial, pre-operative CRT improved survival among patients with potentially curable esophageal or GEJ cancer (mOS of 49.4 months in the CRT–surgery group versus 24.0 months in the surgery group, *p* = 0.003; HR 0.657; 95% CI 0.495 to 0.871) [3,61]. Among patients with adenocarcinoma, mOS of the neoadjuvant CRT-surgery and surgery groups were 43.2 months (24.9–61.4) and 27.1 months (13.0–41.2), respectively (HR 0.73, 95% CI 0.55–0.98; *p* = 0.038). According to the FLOT4 trial, OS was higher in the FLOT group compared to the epirubicin/cisplatin/fluorouracil or epirubicin/cisplatin/capecitabine (ECF/ECX) group (HR 0.77; 95% CI 0.63–0.94; mOS 50 months [38.33 to not reached] vs. 35 months [27.35–46.26]) [6]. A recent report of the NeoAegis study suggested that the MAGIC regimen was similar in efficacy compared to CROSS [62]. In this NeoAegis study, with a median follow up of 24.5 months, the second futility analysis reported 143 deaths including 70 and 73 deaths in the CROSS and MAGIC arms, respectively. In the CROSS and MAGIC arms, the 3-year estimated survival probabilities were 56% (95% CI 47–64) and 57% (95% CI 48–65), respectively (HR 1.02; 95% CI 0.74–1.42). Considering that data were notable for non-inferiority, the DSMB recommended closure of recruitment in December 2020 [62]. Given that the FLOT4 study demonstrated improved OS with FLOT compared to MAGIC, by extension this implies FLOT is also superior to CROSS. However, the ongoing ESOPEC study compared FLOT to CROSS directly has completed accrual, and results are awaited to confirm this outcome [63]. Since then, the role of other modalities such as ICI and targeted therapies in the peri-operative/adjuvant setting has been investigated building on either the CROSS backbone or the perioperative chemotherapy approach [64].

The first report of many ongoing phase 3 studies evaluating ICIs perioperatively were results of CHECKMATE-577, a global, phase 3, randomized, double-blind placebo-controlled trial that evaluated nivolumab for adjuvant use of patients with stage II/III esoSCC or AC (60%) or GEJ carcinomas (40%) irrespective of PD-L1 status was recently reported [65]. Eligible patients had received neoadjuvant CRT and underwent surgical resection (R0, performed within 4–16 weeks prior to randomization) with evidence of residual pathologic disease ≥ypT1 or ≥ypN1. Median follow-up was 24.4 months (range 6.2–44.9), and geographical regions included Europe (38%), the US and Canada (32%), Asia (13%), and rest of the world (16%). With respect to histology, 71% of patients had AC, and 29% had SCC. A total of 794 patients were randomized in a 2:1 ratio to receive either nivolumab (*n* = 532) or placebo (*n* = 262), with a total treatment duration of up to 1 year. In this study, the median DFS, the primary endpoint, was 22.4 months (95% CI 16.6–34.0) in the nivolumab group and 11.0 months (95% CI 8.3–14.3) in the placebo group, respectively (HR 0.69, 96.4% CI 0.56–0.86, *p* = 0.0003). Therefore, nivolumab showed superior DFS with a 31% reduction in the risk of recurrence or death and a doubling of median DFS versus placebo. However, the analysis of DFS by subgroups showed that the DFS favored nivolumab especially in patients with squamous histology (median DFS of 29.7 months in the nivolumab group compared to 11.0 months in the placebo group, HR 0.61) as opposed to AC (HR 0.75, 95% CI, 0.59–0.96) as well as those with esophageal carcinoma (median DFS 24 months in the nivolumab group compared to 8.3 months in the placebo group, HR 0.61) as opposed to GEJ carcinoma (HR 0.87, 95% CI 0.63–1.21). With respect to safety, the majority of TRAEs were grades 1 and 2. Grades 3–4 toxicities were reported in 183 patients (34%) of the nivolumab group and 84 patients (32%) of the placebo group. The FDA approved nivolumab for this indication (20 May 2021) and received category 1 recommendation in the NCCN guidelines. Updated analysis by PD-L1 status demonstrated the benefit pronounced in the subgroup of patients with PD-L1 CPS ≥ 5 28–8 assay, with mDFS of 29.4 months versus 10.2 months (HR 0.62 SS) as compared to PD-L1 CPS < 5, showing no statistically significant difference in mDFS of 16.3 months versus 11.1 months (HR 0.89, NS). Moreover the outcomes by PD-L1 by histology have yet to be reported [66]. However, given the experience with the utility of PD-L1 as a predictive biomarker in the metastatic setting, as well as in early studies suggesting the same [67,68], as well as the subgroups that have been shown here in CHECKMATE-577, it will be important to evaluate these data by PD-L1 and histology (SCC vs. AC), in addition to evaluating the OS data with longer follow up to determine which patients should be treated with ICIs perioperatively. The latter point is important, given the updated report showing PFS2 showing substantially diluted benefit compared to DFS (HR 0.77, 95% CI 0.60–0.99) [66]. Using the same data, PFS2 was defined as the time point of progression on first-line palliative therapy if patients recurred, and patients were censored if they had not yet progressed on first-line therapy or had not recurred. This can be considered an early look at what could be anticipated for OS, which emphasizes priority to identify, likely through PD-L1 testing, which patients are best served to receive 1 year of adjuvant nivolumab, and which are not.

## 7. Hyper-Progression

It should be noted that convincing evidence regarding the entity defined as hyper-progressive disease (HPD) among patients with GEC who have been treated with ICI is currently lacking in prospective studies. HPD is defined as rapid progression with dramatic acceleration of disease trajectory, with expansive growth that is grossly different from baseline, in patients who receive immune-therapy [69]. The reports of HPD among upper GI cancers are scarce and are speculated on the basis of retrospective data. For example, in a retrospective assessment of 25 patients with GI cancers that defined HPD as tumor growth kinetic ratio (TGKR; defined as TGK on ICI divided by pre-immunotherapy TGK) > 2, five patients met the HPD criteria [70], three of which had neuro-endocrine carcinomas. It should be noted that the data was retrospectively obtained from previously performed phase 1 studies and an inherent bias towards selecting patients with more aggressive disease is possible. Overall, lack of a uniform definition, the retrospective nature of studies and also reports of HPD among patients treated with chemotherapy alone, question the existence of HPD as a phenomenon unique to immune-therapy, which could otherwise be explained by the natural history of disease [69,71]. Most importantly, the optimal way to identify HPD would be with ICI monotherapy versus placebo to evaluate if such crossing of curves is observed, which would suggest that the disease is accelerated by the therapy in a subgroup of patients compared to placebo/best supportive care that represents the natural history of the disease. GEC has two such studies, ATTRACTION-02 and CHECKMATE-577 [53,65]. Neither of these two studies demonstrated the classic ‘yin-yang’ phenomenon which was observed in all the other ICI chemo-free studies when compared to an active chemotherapy control. This suggests that ICI chemo-free is ineffective and similar to providing best-supportive care alone (with added ICI clinical and financial toxicity), but that it is not actually accelerating the disease in and of itself beyond the natural disease course. This also supports the practice to selectively treat patients with ICIs, by PD-L1 testing and/or other, in order to spare patients from ineffective yet toxic and costly therapy.

## 8. Future Directions and Combination Strategies to Improve Immune Engagement

Although ICIs either as monotherapy or in combination with chemotherapy improve clinical outcomes marginally over controls of approximately 10–15% absolute improvement in ORR or DFS, there is much room for improvement of clinical outcomes, particularly in PD-L1 negative patients but also in PD-L1 selected patients. Even in MSI-high tumors, which have the best outcomes with ICI, primary refractory disease is seen in approximately 50% of patients, and many more will eventually develop resistance. Table 2 shows a list of some of the notable ongoing phase 3 studies and select phase 2 studies of GEC both in the first-line metastatic and the peri-operative settings.

Moreover, there is a growing list of predictive biomarkers for GEC in the metastatic setting, including PD-L1, HER-2, FGFR2 [20], FGFR3 [72], MET [22], EGFR [19], and claudin [21], with notable overlap amongst them. Personalizing and prioritizing therapy to optimize outcomes for each individual has been demonstrated in the prospective PANGEA platform study [23]. Given the activity shown with combination of ICI with anti-HER2 antibodies, other anti-receptor tyrosine kinase monoclonal antibodies in patients whose tumors harbor other gene amplifications (i.e., EGFR, MET, FGFR2, etc.) or overexpression (i.e., claudin) may be a novel strategy to overcome immune resistance and increase response rates and survival [73]. Future studies should prospectively evaluate these combinations in a personalized and prioritized fashion for each patient.

## 9. Discussion

ICIs first established FDA indications for second-line and third-line treatment of patients with GECs who have MSI-high and PD-L1 positive tumors, respectively. However, the use of ICIs is rapidly evolving, with recent studies evaluating these agents in the first-line treatment and perioperative settings of GECs. In the first-line metastatic setting, although KEYNOTE-062 failed to show superiority in OS and PFS with pembrolizumab compared to chemotherapy for patients with CPS ≥ 1, this was a smaller study and had low percentage of patients with CPS ≥ 10 (~15–20%) in comparison to the incidence among more recent studies including KEYNOTE-590 (CPS ≥ 10 in ~50% of patients), CHECKMATE-649 (CPS ≥ 5 in ~60% of patients). On the other hand, ATTRACTION-04 did not show survival benefit and it would be interesting to evaluate the incidence of higher PDL1 CPS tumors within that study. Taken together, all the first-line studies suggest that ICI benefits those with higher PD-L1 scores of either CPS ≥ 5 or CPS ≥ 10, and best in combination with chemotherapy. These cut-offs are likely adequate to identify those patients who will derive significant benefit from ICIs, and in fact the optimal cut-off is likely higher. This observation is reflected in the NCCN guidelines for nivolumab and pembrolizumab (as well as by the recent EMA decisions for pembrolizumab based on KEYNOTE-590 and nivolumab based on CHECKMATE-649). It is notable that most MSI-high tumors have high PD-L1 expression (65% with CPS ≥ 5, 95% with CPS ≥ 1), and by default would be eligible for ICI therapy in the first-line setting. MSI-high patients derived the most benefit in every study examined, including CHECKMATE-649 [33,42]. In fact, in CHECKMATE-649, the unstratified HR for OS with nivolumab plus chemotherapy versus chemotherapy alone was 0.33 (95% CI 0.12–0.87) for patients with MSI-high tumors and 0.73 (0.62–0.85) for microsatellite stable tumors in patients with a PD-L1 CPS ≥ 5, and 0.37 (0.16–0.87) for patients with MSI-high tumors and 0.80 (0.71–0.91) for microsatellite stable tumors overall irrespective of PD-L1 status [42].

Importantly, patients with absent or low PD-L1 expression show minimal to no response with the addition of ICI monotherapy or in combination with chemotherapy, and no significant improvement in PFS or OS, and have worse outcomes compared to relevant control treatments. As such, in our practice patients with low/negative PD-L1 tumors are not considered for upfront ICI alone or with chemotherapy. Rather, studies evaluating combinations and newer immunotherapeutic approaches are warranted to try to better harness the immune system in these primary refractory and immune ‘cold’ tumors. Also, there is a pressing need to identify biomarkers with higher PPV of response to ICI among these patients since the ORR is still only ~10–15% above controls with ICIs, even in PD-L1 enriched populations with or without chemotherapy. Such predictive tools would help direct where it is adequate to treat with ICI alone or ICI with chemotherapy, and where something else is necessary [26,74,75,76,77].

Special considerations should be made before making any cross-trial comparisons since these trials vary in biomarker assessment and PD-L1 diagnostic antibodies, as well as many other factors. First, there is lack of consensus to define PD-L1 positivity. In most early studies, patients with PD-L1 CPS ≥ 1 were considered to be PD-L1 positive. However, as reviewed above, different studies have considered varying levels of CPS as the threshold for defining PD-L1 positivity (Table 3), and generally the higher the threshold of positivity, the more predictive of benefit (better PPV), at the expense of NPV; while the lower the cut-off, the better the NPV at the expense of PPV. Confusingly, while some studies use CPS for PD-L1 expression scoring, some other trials have used TPS as their scoring system [37,46,51,76]. Moreover, the use of different antibodies, such as 22C3 pharmDx assay in KEYNOTE studies, 28-8 PharmDx assay in CHECKMATE and ATTRACTION studies, and 73-10 PharmDx assay in JAVELIN studies, further complicates the interpretation of these results [35,37,46,51,76]. Considering a CPS ≥ 1, CHECKMATE-649 reported an 80–85% PD-L1 positivity using the 28-8 PharmDx assay, while different KEYNOTE studies have reported a PD-L1 CPS ≥ 1 positivity rate of about 60% using the 22C3 assay. Using a CPS ≥ 5 as the threshold, CHECKMATE-649 reported almost 60% PD-L1 positivity (28-8 assay), while the KEYNOTE-061 study reported an approximate 30% rate of PD-L1 CPS ≥ 5 positivity (22C3 assay) [78]. It is notable that a few studies have directly compared the different antibodies and have generally reported concordance between them, which is reassuring that the difference in antibodies does not seem to be a major explanation for inter-trial discordance in PD-L1 positivity incidence [79,80]. Importantly, even with the same antibody and scoring system, there has been stark contrasts between KEYNOTE studies. Considering CPS ≥ 10 as the threshold, several early KEYNOTE studies reported a 15–20% rate of positivity (22C3 assay), while the more recent KEYNOTE-590 study reported a 50% positivity rate, which is considerably higher. In fact, pre-screening and preferentially enrolling patients with higher PD-L1 expressing tumors in more recent studies due to the knowledge gained from the earlier studies could be a potential source for selection bias in more contemporary trials, artificially inflating the true incidence of PD-L1 positivity that would be otherwise observed in all-comers.

Several other considerations should be made before concluding the utility of ICIs based on the aforementioned first-line setting trials. Notably, the different proportion of Asian patients among these trials could have potentially affected the response rates to ICIs via different tumors and underlying host biology, which may at least partially explain the ORIENT-15 and JUPITER-06 results in contrast to KEYNOTE-590, CHECKMATE-648, and ESCORT-1 SCC studies. As noted previously, Asian patients with GEC who receive chemotherapy have been historically shown to have higher survival rates than non-Asian patients. Overall, the same trend seems to be true in case of ICIs for unknown reasons and should be considered when comparing trials with different composition of Asian and non-Asian patients.

Moreover, the impact of using cytotoxic agents together with ICI on T-cell activity and clonal expansion remains to be elucidated, although the combination ICI with chemotherapy seem to be synergistic [81]. As noted in KEYNOTE-062 MSI-high cohort, pembrolizumab alone induced a clear benefit compared to chemotherapy alone, and while there was no significant benefit from additional chemotherapy in the combination arm in terms of OS, this was a small subset without power to evaluate this question; importantly there did appear to be an improved PFS with the combination with estimated 36 month PFS rates of 55% with pembrolizumab + chemotherapy versus 25% with pembrolizumab alone versus 15% with chemotherapy alone, albeit with small numbers [33]. Therefore, whether to start chemo-immunotherapy versus immunotherapy alone for first-line treatment of MSI-high tumors remains an open question. The evidence in chemo-immunotherapy studies such as KEYNOTE-062 and CHECKMATE-649 show benefit with combination, but the monotherapy and/or chemo-free arms of KEYNOTE-062 and CHECKMATE-649, as well as KEYNOTE-177 in colorectal cancer, suggest that there is at least a subset of patients with MSI-high tumors that could be adequately treated with ICI therapy alone. Extending this further to microsatellite stable patients, there is a small subset that seem to be adequately treated with ICI monotherapy as seen from the KEYNOYE-062 study as well as later-line monotherapy ICI studies. Further studies should be conducted to better direct ICI therapy for GEC patients towards either ICI monotherapy or dual ICI therapy, chemo-immunotherapy combination, versus other novel therapies for those unlikely to benefit from current ICI therapeutic strategies. Another open question is whether the backbone chemotherapy in combination chemo-immunotherapy could potentially influence the primary endpoints of the trials. For example, some preclinical data suggest that cycles of 5FU can impair cytotoxic T cell effector functions [82,83]; however, the exact influence of different chemotherapy agents that could impact tumor microenvironment and potential response to ICI remains to be elucidated [84,85].

## 10. Conclusions

Immune-checkpoint inhibition has shown promise in the treatment paradigm of GEC, as more trials provide data on their safety and efficacy profiles. However, given that these drugs do increase grade >3 toxicity by 10–15% over controls, along with higher all-grade toxicity, coupled with the financial burden imposed on health care systems and patients, identifying who should and should not get ICI therapy as monotherapy, dual ICI therapy, or in combination with chemotherapy is paramount. According to the current data, first-line ICI use appears to most benefit patients with high PD-L1, a minimum CPS ≥ 5, and likely better is CPS ≥ 10. While low PD-L1 expression status has a good NPV for response to ICI, merely having a CPS ≥ 5 or CPS ≥ 10 still has a low PPV of benefit, improving ORR by ~10–15% over controls. Therefore, there is an ongoing search for identifying reliable biomarkers of response to ICI among these PD-L1 enriched patients. Other open questions include the nature and potential impact of cytotoxic agents on T-cell activation and clonal expansion and the possible role of combination strategies of targeted and novel immunomodulatory therapies in improving immune engagement and augmenting response to immune checkpoint inhibition with anti-PD-1/PD-L1 antibodies.

## Figures and Tables

**Table 1:** Summarization of the trials evaluating the safety and efficacy of immune-checkpoint inhibitors for the treatment of gastro-esophageal carcinoma in different settings. Details of chemotherapy regimen, immune-checkpoint inhibitor and absence/presence of a placebo and number of patients in each arm is provided in the column designated as study arms. (KN: KEYNOTE; CM: CHECKMATE; ATTRCN: ATTRACTION; JVLN: JAVELIN; 1L: first-line; 2L: second-line; 3L: third-line; SCC: squamous cell carcinoma; EsoSCC: esophageal squamous cell carcinoma; GEJAC: Gastro-esophageal junction adenocarcinoma; GC: gastric cancer; Ab: antibody; OS: overall survival; HR: hazard ratio; CPS: combined positive score; TPS: tumor positive score; NS: not significant; ORR: objective response rate; Nivo: nivolumab; Ipi: ipilimumab; Pembro: pembrolizumab; NR: not reported; pts: patients).

Trial	Line of Treatment	Year	Size	Study Arms	SCC/AC	EsoSCC/GEJAC/GC	Asian (%)	Biomarker/Histo	Biomarker Incidence	Ab	OS HR
**3L Studies**											
KN-059	3L (cohort 1)	2018 [28]	259	Pembro only (*n* = 259)	0/100	0/51.4/48.3	15.8	CPS ≥ 1	57.1%	22C3	ORR 11.6%
ATTRCN-02	3L	2017 [34]	493	Placebo (*n* = 163) vs. nivo (*n* = 330)	0/100	0/5.5/62.5	100	TPS ≥ 1	13.5% (among 192 pts)	28-8	0.63
JVLN-300	3L	2018 [35]	371	Paclitaxel or irinotecan (*n* = 186) vs. avelumab (*n* = 185)	0/100	0/30/70	25	TPS ≥ 1	26.8% (among 317 pts)	73-10	1.1 (NS)
**2L Studies**											
KN-061	2L	2018 [36]	592	Paclitaxel (*n* = 296) vs. pembro (*n* = 296)	0/100	0/31/69	30	CPS ≥ 1	66.7%	22C3	0.82 (NS)
KN-181	2L	2020 [37]	628	Paclitaxel, docetaxel or irinotecan (*n* = 314) vs. pembro (*n* = 314)	64/36	64/36/0	39	CPS ≥ 10	35.3%	22C3	0.69
ATTRCN-3	2L	2019 [38]	419	Paclitaxel or docetaxel (*n* = 209) vs. nivo (*n* = 210)	100/0	100/0/0	96	TPS ≥ 1	TPS ≥ 1.48%, TPS ≥ 5.35%, TPS ≥ 10.29%	28-8	0.77
RATIONALE-302	2L	2021 [39]	512	Paclitaxel, docetaxel or irinotecan (*n* = 256) vs. tislelizumab (*n* = 256)	100/0	100/0/0	79	CPS ≥ 10	30.6%	SP263	0.7
**1L Studies**											
KN-062	1L	2020 [40]	763	Cisplatin with 5FU or capecitabine plus placebo (*n* = 250) vs. cisplatin with 5FU or capecitabine plus pembro (*n* = 257) vs. pembro alone (*n* = 256)	0/100	0/30/70	24.5	CPS ≥ 1	100%	22C3	0.85 (NS) for chemo + pembro vs. chemo, and 0.85 (NS) for chemo + pembro vs. chemo in CPS ≥ 10
ATTRCN-4	1L	2020 [41]	724	Oxaliplatin with S-1 or capecitabine plus placebo (*n* = 362) vs. oxaliplatin with S-1 or capecitabine plus nivo (*n* = 362)	0/100	0/0/100	100	TPS ≥ 10	16%	28-8	0.9 (NS)
CM-649	1L	2020 [42]	1581	Oxaliplatin with 5FU and leucovorin or oxaliplatin with capecitabine plus nivo (*n* = 789) vs. oxaliplatin with 5FU and leucovorin or oxaliplatin with capecitabine (*n* = 792)	0/100	0/30/70	24	CPS ≥ 5	CPS ≥ 5.60%, CPS ≥ 1.82%	28-8	0.71 in CPS ≥ 5, 0.77 in CPS ≥ 1
KN-059	1L (cohort 2)	2019 [43]	25	Cisplatin with 5FU or capecitabine plus pembro (*n* = 25)	0/100	NR	68	CPS ≥ 1	64%	22C3	ORR 60%
KN-059	1L (cohort 3)	2019 [44]	31	Pembro only (*n* = 31)	0/100	NR	48.4	CPS ≥ 1	100%	22C3	ORR 25.8%
ORIENT-16	1L	2021 [45]	650	Capecitabine and oxaliplatin plus placebo (*n* = 323) vs. capecitabine and oxaliplatin plus sintilimab (*n* = 327)	0/100	0/18.5/81.5	100	CPS ≥ 5	61%	NR	0.76, 0.66 in CPS ≥ 5
KN-590	1L	2020 [46]	749	Cisplatin and 5FU plus pembro (*n* = 373) vs. cisplatin and 5FU plus placebo (*n* = 376)	73/27	73/27/0	52	CPS ≥ 10	CPS ≥ 10.50%, SCC + CPS ≥ 10.52%	22C3	0.57 in SCC + CPS ≥ 10, 0.62 in CPS ≥ 10, 0.72 in SCC, 0.73 in Asians
CM-648	1L	2021 [47]	970	Cisplatin and 5FU (*n* = 324) vs. cisplatin and 5FU plus nivo (*n* = 321) vs. nivo plus ipi (*n* = 325)	100/0	100/0/0	70	TPS ≥ 1	49%	28-8	0.54 for nivo + chemo vs. chemo in TPS ≥ 1, 0.64 for nivo + ipi vs. chemo in TPS ≥ 1
ESCORT-1^st^	1L	2021 [48]	596	Cisplatin and paclitaxel plus placebo (*n* = 297) vs. cisplatin and paclitaxel plus camrelizumab (*n* = 298)	100/0	100/0/0	NR	NR	NR	NR	0.7
ORIENT-15	1L	2021 [49]	659	cisplatin and 5FU or cisplatin and paclitaxel plus placebo (*n* = 332) vs. cisplatin and 5FU or cisplatin and paclitaxel plus sintilimab (*n* = 327)	100/0	100/0/0	97	CPS ≥ 10 TPS ≥ 10%	CPS ≥ 10 57.8% TPS ≥ 10% 36.1%	NR	0.62 for all patients, 0.63 in CPS ≥ 10
JUPITER-06	1L	2021 [50]	514	Cisplatin and paclitaxel plus placebo followed by placebo maintenance (*n* = 257) vs. cisplatin and paclitaxel plus toripalimab followed by toripalimab maintenance (*n* = 257)	100/0	100/0/0	100	CPS ≥ 1	CPS ≥ 1 78% CPS ≥ 10 41.2%	NR	0.58, 0.61 in CPS ≥ 1
KN-811	1L	2021 [51]	264	5FU and cisplatin and trastuzumab or capecitabine and oxaliplatin and trastuzumab plus pembro (*n* = 133) vs. 5FU and cisplatin and trastuzumab or capecitabine and oxaliplatin and trastuzumab plus placebo (*n* = 131)	0/100	0/30/70	30	CPS ≥ 1	86%	22C3	ORR 74.4% in pembro + chemo vs. 51.1% in chemo + placebo
MAHOGANY	1L (cohort A)	2021	43	Margetuximab plus retifanlimab (*n* = 43)	0/100	0/41.9/58.1	44.2	CPS ≥ 1	100%	NR	Tumor shrinkage 85.7%
JVLN gastric 100	1L maintenance	2021 [52]	499	5FU and oxaliplatin and leucovorin or capecitabine and oxaliplatin as maintenance (*n* = 250) vs. avelumab as maintenance (*n* = 249)	0/100	0/28.8/71.2	29.8	TPS ≥ 1% CPS ≥ 1	TPS ≥ 1% 12.5%, CPS ≥ 1 64.3	73-10 and 22C3	0.91 (NS)

**Table 2:** Notable ongoing phases 2 and 3 studies for the first-line treatment of gastro-esophageal cancer (GEC) both in the metastatic and peri-operative settings. The contents of this table are retrieved from clinicaltrials.gov (1L: first-line; CAPOX: capecitabine/oxaliplatin; FOLFOX: 5-fluorouracil/oxaliplatin; SOC: standard of care; GEJ: gastro-esophageal junction; GC: gastric cancer; XP: capecitabine/cisplatin; FP: 5-fluorouracil/cisplatin).

Study Name/Title	Study Description/Arms and Intervention	Histology/Setting
**1L, Locally Advanced Unresectable or Metastatic Setting**		
LEAP-014 This is a phase 3, randomized study that evaluates the efficacy and safety of Pembrolizumab + Lenvatinib in combination with chemotherapy compared with standard of care	There will be 2 parts to the study: the Safety Run-in (Part 1) and the main study (Part 2). In part 1, participants will be treated with pembrolizumab plus lenvatinib plus chemotherapy. In part 2, participants (not including those participating in part 1) will be treated with pembrolizumab plus lenvatinib plus chemotherapy or pembrolizumab plus chemotherapy.	1L, metastatic squamous cell carcinoma of the esophagus
LEAP-015 This is a phase 3, randomized study that evaluates the efficacy and safety of Lenvatinib + Pembrolizumab in combination with chemotherapy compared with standard of care	This study consists of 2 parts: In part 1, participants will be treated with lenvatinib plus pembrolizumab and chemotherapy (either CAPOX or mFOLFOX6), and then followed for dose-limiting toxicities for 3 weeks. In part 2, participants will be randomly enrolled to receive either lenvatinib in combination with pembrolizumab and chemotherapy (CAPOX or mFOLFOX6) or chemotherapy alone (CAPOX or mFOLFOX6).	locally advanced unresectable or metastatic HER2-negative GEJ adenocarcinoma
SKYSCRAPER-07 This is a phase 3, randomized study of atezolizumab with or without Tiragolumab (Anti-TIGIT Antibody) in patients with unresectable esophageal squamous cell carcinoma who did not have progression of disease after chemoradiotherapy	In the experimental arm A, participants will receive atezolizumab followed by tiragolumab. In the experimental arm B, participants will receive atezolizumab followed by tiragolumab matching placebo, and in arm C (placebo comparator), participants will receive Tiragolumab placebo + Atezolizumab placebo. Participants will receive matching placebos to tiragolumab and atezolizumab.	Unresectable squamous cell carcinoma of the esophagus, without progression after definitive chemoradiation
KEYNOTE-859 This is a phase 3, randomized study of Pembrolizumab in combination with chemotherapy versus placebo in combination with chemotherapy among patients with HER2-negative, previously untreated, unresectable/metastatic GC or GEJ adenocarcinoma	In the experimental arm, the participants will receive pembrolizumab + physicians’ choice of either cisplatin and 5FU OR oxaliplatin + capecitabine. Participants who complete 35 administrations or achieve a complete response (CR) but progress after discontinuation can initiate a second course of pembrolizumab for up to 17 cycles (approximately 1 additional year).	1L, untreated, unresectable or metastatic HER2-negative GC or GEJ adenocarcinoma
KEYNOTE-811 This is a phase 3, randomized trial comparing Trastuzumab plus chemotherapy and Pembrolizumab with Trastuzumab plus chemotherapy and placebo as first-line treatment in participants with HER2-positive advanced GC or GEJ adenocarcinoma	Pembrolizumab or placebo will be administered in addition to trastuzumab, in the beginning of each cycle. For the global cohort, SOC chemotherapy will constitute either FP (Cisplatin + 5-FU) or CAPOX. The Japan cohort, will be treated with SOX chemotherapy consisting of S-1 (tegafur, 5-chloro-2,4-dihydroxypyridine [CDHP], and potassium oxonate [Oxo] and oxaliplatin).	locally advanced unresectable or metastatic HER2-positive GC or GEJ adenocarcinoma
MAHOGANY This is a phase 2/3 study that evaluates Margetuximab in combination with INCMGA00012 and chemotherapy or MGD013 and chemotherapy for the treatment of metastatic or locally advanced, HER2-positive GC or GEJ cancer	In cohort A (single arm) the safety and efficacy of margetuximab plus INCMGA00012 will be evaluated. In cohort B part 1 (including 4 arms), patients will be randomly assigned to: margetuximab plus chemotherapy plus INCMGA00012 arm, margetuximab plus chemotherapy plus MGD013 arm, margetuximab plus chemotherapy arm, and trastuzumab plus chemotherapy arm. In cohort B part 2, a checkpoint inhibitor (INCMGA00012 or MGD013) will be selected and evaluated in another randomized 2 arm cohort, consisting of margetuximab plus chemotherapy plus INCMGA00012 or MGD013, or trastuzumab plus chemotherapy.	locally advanced unresectable or metastatic HER2-positive GC or GEJ adenocarcinoma
**1L Locoregional/Resectable Setting**		
KEYNOTE-585 This is a phase 3, randomized study of Pembrolizumab in combination with chemotherapy (XP or FP) versus placebo plus chemotherapy (XP or FP) as neoadjuvant or adjuvant treatment of patients with GC and GEJ adenocarcinoma	In the experimental arms: Neoadjuvant: Prior to surgery, participants receive 3 cycles of pembrolizumab PLUS cisplatin and capecitabine OR cisplatin and 5-FU. Adjuvant: 4 to 10 weeks post-surgery, participants receive 3 cycles of pembrolizumab PLUS cisplatin and capecitabine OR cisplatin and 5-FU, followed by pembrolizumab monotherapy for up to 11 additional cycles. This study also includes pembrolizumab + FLOT cohort and a placebo + FLOT cohort, both in the neoadjuvant and adjuvant settings.	localized GC or GEJ adenocarcinoma
MATTERHORN This is a randomized, phase 3 study of neoadjuvant Durvalumab and FLOT chemotherapy followed by adjuvant Durvalumab for the treatment of resectable GC and GEJ Cancer	This study evaluates the efficacy of treatment with durvalumab or placebo combined with FLOT given before surgery (neoadjuvant setting) and durvalumab or placebo therapy combined with FLOT chemotherapy after surgery (adjuvant setting)	GC or GEJ adenocarcinoma with resectable disease
ATTRACTION-05 This is a randomized study in patients with GC undergoing postoperative adjuvant chemotherapy	In the experimental (nivolumab) group, patients will get: Nivolumab + chemotherapy (S-1 therapy or CAPOX therapy is determined by the investigator). In the placebo comparator, patients will get placebo + chemotherapy.	Adjuvant, histologically confirmed GC, status post R0 resection
DANTE This is a randomized phase 2 study of Atezolizumab in combination with FLOT versus FLOT alone in patients with GC or GEJ cancer	Eligible patients will be randomized to two arms: Arm A: patients will receive atezolizumab + FLOT before undergoing surgery. Following surgery, patients will receive additional cycles of atezolizumab + FLOT followed by atezolizumab alone. Arm B: FLOT alone: Patients will receive FLOT alone before surgery. Following surgery, patients will receive additional cycles of chemotherapy alone.	adenocarcinoma of the GEJ or GC (cT2, cT3, cT4, any N category, M0, or any T, N+, M0) that is considered medically and technically resectable
EA2174 This is a phase 2/3 study of peri-operative Nivolumab with or without Ipilimumab for treatment of locoregional esophageal and GEJ adenocarcinoma	Arm A: Patients will be treated with carboplatin and paclitaxel and undergo radiation therapy as well. Arm B: Patients will be treated with carboplatin, paclitaxel, and radiation therapy as well as nivolumab. Arm C: Patients receive nivolumab only. Arm D: Patients will be treated with nivolumab plus ipilimumab.	esophageal or GEJ adenocarcinoma, staged as T1N1-3M0 or T2-3N0-2M0

**Table 3:** PD-L1 diagnostic antibodies and scoring systems reported to date. Different studies, including KEYNOTES, CHECKMATES, ATTRACTION, JAVELINS, have used different diagnostic antibodies and scoring systems. In addition, these studies have used different positivity incidences based on CPS or TPS. The variability in positivity incidences will result in different NPV and PPV of response to ICI (CPS: combined positivity score; TPS: Tumor positivity score; NPV: Negative predictive value; PPV: positive predictive value).

Therapeutic Antibody/Studies	Diagnostic Antibody	Scoring System	Positivity Incidence	Comments
Pembrolizumab/KEYNOTES	22C3 pharmDx assay	CPS Cut off ≥ 1 ≥10 (or other)	CPS ≥ 1, 50–60% CPS ≥ 10 15–25%	Good NPV, not great PPV; enrich for benefit at higher cut-offs
Nivolumab/CHECKMATE, ATTRACTION	28-8 pharmDx assay	TPS or CPS Cut off ≥ 1% or CPS > 5	TPS ≥ 1 13.5–25% CPS ≥ 1 82% CPS ≥ 5 60% CPS ≥ 10?	Poor NPV TPS Not enriching
Avelumab/JAVELIN	73-10 pharmDx assay	TPS Cut off ≥ 1	10–26.8%	Poor NPV Not enriching
